# Book Reviews

**Published:** 1820-05

**Authors:** 


### CRITICAL ANALYSIS

OF

### RECENT PUBLICATIONS, IN THE DIFFERENT BRANCHES OF MEDICINE AND SURGERY;

### SELECT MEMOIRS, AND HISTORIES OF CASES;

### In the Literature of Foreign Nations.

Πατρίδες ἄρα
Ανδράσιν, οὐ πατραῖς ἄνδρες, ἀγαλλόμεθα.

***Medico-Chirurgical Annuary of the Civil Hospitals and Infirmaries of Paris; or, a Collection of Memoirs and Observations by the Physicians and Surgeons of those Establishments.*** **4to. pp. 636; with fourteen Plates. Paris, 1819.**

[In continuation from page 332.]

***A new Division of Apoplexies.*** **By A. Serres, Chevalier of the Legion of Honour; one of the Physicians of the Hospital La Pitié.** **[In continuation from page 332.]**

In the analysis of the part of this memoir which has already engaged our attention, we have shown that M. Serres considers that compression of the brain is not the cause of the phenomena by which apoplexy is characterized; that simple apoplexy arises from some morbid state of the meninges of the brain solely; and that apoplexy accompanied by paralysis, depends on some lesion of the structure of the brain itself: and hence, that it is possible, during the life of the patient, to know what species of apoplexy is present. If the patient move all his limbs when he is excited, the apoplexy is simple, it has its seat in the meninges of the brain; if hemiplegia exist, or the mouth be drawn to one side, it is the substance of the brain itself that is the seat of the morbid lesion.

We have already taken into consideration the first proposition; and we intended to examine at some length the two latter; but the author opposes a scepticism of the correctness of the observations of others, that breaks the force of the arguments we could oppose to his hypothesis. If we say that dissection has showed the existence of blood effused in the substance of the brain, and collected in a sort of cell formed by the surrounding portion of this organ, when no paralysis had existed; he denies the validity of this last observation: paralysis might have been present, and was overlooked, because we had attached but little importance to what we might regard as a merely negative sign, or we did not assure ourselves of its absence in the state of stupor in which apoplectics generally lie. If, on the other hand, we cite, from observation or literary records, cases where hemiplegia had existed, and no signs of lesion of the substance of the brain were perceptible after death; M. Serres replies, that the non-discovery of this lesion might have depended on want of accuracy in making the examination. He thus places an impregnable barrier between his system and any arguments we can adduce against it; and it is only those who shall hereafter investigate the subject with the views above indicated, who will be permitted to pass that barrier, and engage in the contest on fair ground.

The connexion of apoplexy with disorder of the stomach and intestines, has been long, and often, remarked by physicians, and it has been supposed that, in many cases, the latter affection was the exciting cause of the former. This doctrine is also opposed by M. Serres; and he firmly believes that he has positively refuted it. When we state that (as it appears from the memoir before us), his conclusion has been founded on only seventeen cases, presenting, at best, but *negative arguments* against the doctrine he says he has refuted, we shall give such evidence of—(we will use the term)—a *passion* for generalization, as will make prudent men regard even his observations with much reserve. We shall designate those points in the seventeen cases above alluded to that relate to our present object. Seven apoplectics were brought into the Hotel Dieu in a dying state, and ceased to live at from thirty to fifty hours after the attack. *No medicine was administered.* Neither the stomach nor intestines presented any signs of inflammation. Of *ten* others, whom he also dissected, *four* had tartar emetic given to them, in doses of six grains, several times repeated, without vomiting having been produced; *eight* were treated by purgative glysters, from a convulsive closure of the jaws having prevented deglutition. In the first four, he found the stomach and the commencement of the jejunum in a state of the most intense phlogosis; the mucous membrane might be detached in layers, leaving bare the muscular coat; some blackish spots were dispersed here and there in the small intestine: the peritoneum was partially inflamed in both. In those who, not having been able to swallow any thing, had been treated by purgative glysters, the stomach and small intestines presented no sensible lesion; but the whole of the great intestines, from the valve of the coecum to the termination of the rectum, were contracted; the internal membrane dark and invested with pellicles, floating in their cavity, of from an inch to eighteen lines in length; the muscular and peritoneal coats partook of this violent irritation. M. Serres then adds, "I conclude, from these facts, that the inflammatory traces presented in these bodies were effects produced by the action of medicines, and entirely foreign to the development of the apoplexy: since, 1°, if the patients died before any medical measures had been resorted to, the stomach and intestines were in the ordinary state, without the slightest sign of inflammation; 2°, if irritants had been employed; inflammation was developed in the stomach and the small intestines, when emetics and purgatives had been given; in the large intestines solely, when purgative glysters had been employed.

"Thus, inflammation was manifested where irritants had been applied; and there was no inflammation when their use was not resorted to. The inflammatory traces observed in the alimentary canal of apoplectics cannot then be regarded as a cause of that disease." Here M. Serres forms a positive conclusion of general application, from postulates which are merely negative; but analogy, he considers, supports his inferences, from the evidence of acute gastritis and enteritis being every day witnessed, without being accompanied by apoplexy, and from all the epidemic mucous fevers observed in Europe having presented irritations, more or less intense, of the stomach and the small intestines, without apoplexy accompanying their progress. The arguments drawn from gastritis and enteritis are positive, and somewhat powerful, but not decisive; because, as we have already remarked, certain causes will produce certain effects in the animal body at one time or in one person, that are not produced at other times or in other persons. The argument drawn from the existence of inflammation of the mucous membrane of the alimentary canal in fevers, is not so forcible; because it remains to be proved whether or not that inflammation were itself sympathetic of irritation in some other part, and this being the case, whether it might not excite different effects on other organs, the brain for example, from those it produces when it is idiopathic. We must also know in what the difference between the states of stupor or coma of the patients of those fevers, and that of apoplexy, consists, before we can say that *apoplexy* has not accompanied the progress of gastric fever. We know not how M. Serres has got over those difficulties, or whether he has discerned them; but he immediately concludes, that those authors have erred "who have attributed the development of certain apoplexies to the irritations, more or less intense, which they have found after death in the stomach, the small intestines, and the large intestines, of *apoplectics.* We may then regard as established on facts the proposition, that *all apoplexies have their primitive seat in the cerebrum, the cerebellum, or their envelopments.*"

The cases alluded to in our former article on this subject, and which form the bases of his new division of apoplexies, are related in detail by M. Serres. They occurred in a public hospital, were most of them observed by other physicians during the life, and after the death, of the subjects, and appear to have been investigated throughout with great care. Supposing that the observations of M. Serres were accurately made; it must be considered, that a hundred cases, showing that in apoplexy accompanied by paralysis there was always lesion of the substance of the brain; and that, when there was apoplexy without paralysis, no such lesion was present; without any other cases furnishing different results; present such grounds for the inferences he has drawn from them, of general application to these maladies, that we could hardly refuse to admit the truth of his conclusions, were it not that we daily see instances of various kinds of lesion of the same parts of the brain as those which formed the seat of the coagulum of blood in the cases of M. Serres: as morbid tumors, in which there is no appearance whatever of the natural structure of the brain, abscesses, pultaceous disorganization, &c. without having been accompanied with hemiplegia. If the disorganization of the brain attending the collection of a coagulum of blood in the substance of the brain be the cause of the paralysis which accompanies apoplexy, independent of any pressure it may exert on the brain, we can discern no reason why the disorganizations above designated should not produce paralysis without apoplexy. We are conscious that this last objection is not a strictly precise one, and therefore not very forcible: there is a difference between the morbid state attending the two series of disorganizations, that is, those presenting a collection or coagulum of blood, and those presenting tumors, abscesses, &c.; and therefore there may be reasons why paralysis should arise from the one and not from the other, though we cannot discern them.

Although the doctrine of M. Serres may not be admitted to be true, his observations must lead physicians in general to new investigations of this subject, consonant to his views and opinions; and it seems probable, that no considerable period will elapse before the truth or falsity of his theory be ascertained.

### *Some Observations on the Use of Opium in Bilious Fevers.* By M. Husson, Physician to the Hotel Dieu.

On visiting, in 1804, the hospital for prisoners at Toulon, M. Husson regarded with much interest, in the records of the medical practice in that hospital, the shortness of the duration of "*bilious fevers*," when treated with opium, by M. Hernandez; and, on comparing the records of these cases with that of the same number of other patients in the same hospital, living under similar physical and moral influences, he determined to resort himself to the use of that remedy in any cases of "bilious fever" that might come under his care. Whether or not M. Husson made any enquiry respecting the relative proportion of *deaths* in the patients under the care of M. Hernandez, to that under the treatment of the other physician, who used evacuating means, he does not say: we suspect not. The shortness of the duration of the disease is all that, it appears, occupied his attention. Soon after his return to Paris, he had three patients affected with "well-characterized bilious fever" come under his care at the Hotel Dieu; he administered to the whole of them, on his first visit, a drachm of the liquid laudanum of Sydenham, at once. "In two of these patients the gastric symptoms were dissipated on the next day; they continued in the third. He administered to the whole of them half-a-drachm of laudanum at once, on this day. The two former were able to leave the hospital on the fourth day after their entry; whilst the third patient, who did not derive the same benefit from the opium, still had fever, and was cured only by the use of the evacuating method, that is to say, tartarized antimony and purgatives." After this he resumed, and continued for several years, the evacuating method; until the summer of 1815, when the great number of patients with *bilious fever* who entered the Hotel Dieu, again excited his particular attention to the practice of M. Hernandez. He, however, resorted to it in but three cases. In two of them the results were equally favourable with those of two of the former patients; but the third "had an accident, which was not remedied in time, and she (a woman aged twenty-five, of a strong constitution,) died of *un narcotisme très-marqué*, although she had taken only half the dose of opium administered to one of the two others." One drachm of Sydenham's laudanum was the quantity administered to this unfortunate woman. We regret, with M. Husson, that she was neglected during the rest of the day after taking the opium; or, as he says, emetics aud vegetable acids might have remedied the accidents it produced. This is a point for separate consideration. With respect to the merits of the medicine, it appears that, on this occasion, two patients recovered from their disease, by its influence, more rapidly than they might have done under the application of other means: whether or not their lives were preserved by it, is a matter of doubt; but that it killed the third, is morally certain. M. Husson may practise his art for many years, before he can counterbalance this act, by an instance of the positive preservation of the life of another equally valuable member of society.

*Case of Hematemesis, or Vomiting of Blood, complicated with Hemoptysis.* By M. Husson.

A woman, thirty-two years of age, subjected to constant domestic troubles, from moral disagreements with her associates, received two violent blows on the epigastrium, one a few months after the other: her health languished remarkably after each, but especially after the latter. She had obtuse pain about the stomach, the functions of this organ were imperfectly performed, and menstruation became irregular. At the end of three months the pain extended to the chest. Vomiting and expectoration of blood soon afterwards took place. All food was rejected from the stomach, mingled with blood. She entered the Hotel Dieu on the 1st of March, 1815, about two months after the occurrence of the latter circumstances. For a month previous to her entry into the hospital, the only food she had taken was water sweetened with sugar, and aromatized with orange-flower water. Leeches, cupping, vesicatories, and opiate plasters, were in succession applied to the epigastrium, which was very painful; frictions with laudanum were tried, but "the patient could not bear them, any more than the warm bath." "It was attempted, without utility, to administer inwardly, *etherial emulsions,(!) opium,(!!) musk,(!!!)* camphor, &c. under diverse forms: these medicines, as well as milk and barley-water, given as aliments, were rejected by vomiting, always with a considerable quantity of blood. Glysters of broth were then alone administered, several times a-day. The patient, fatigued by the use of all the means which art has at its disposal in such a case, abandoned herself to nature. The 15th of May, 1815, she was almost entirely well, and left the hospital." We think we should abandon ourselves to nature, too, if we should fall down sick in the streets of Paris, and be carried to the Salles Sainte-Monique or de la Creche, at the Hotel Dieu.

*Case of Aneurism of the Heart.* By M. Husson.

A woman, seventy years of age, who ceased to menstruate at fifty-two, had for twenty years experienced an habitual cough and very great difficulty of breathing, fatigue on using exercise, and sense of suffocation on going up stairs. These symptoms, after having been stationary for some time, had much increased in severity after a fit of anger. She entered the Hotel Dieu on the 15th of February, 1815. The face was bluish, the respiration difficult; she had a sensation of imminent danger of suffocation; the voice was hoarse; *the external jugular veins, and all their sub-cutaneous ramifications, were varicose to an enormous degree:* they had been so for six years. She died on the 25th of the same month.

On dissection, "the volume of the heart was found a little more considerable than in the natural state; the pericardium contained a small quantity of serous fluid; but the parietes of the *left ventricle* were so much thickened that no cavity existed in it." This case of hypertrophy of the heart (we cannot discern why it is called *aneurism*) is very interesting; or, more properly speaking, it would be very interesting, if M. Husson had expressly stated that the parietes of the *right ventricle* were *not thickened;* but he only lets us suppose this to have been the case, by his neglecting to say any thing expressly on this point, and stating precisely that it existed in the *left ventricle.* Dilatation of the jugular veins has been commonly remarked in cases of thickening of the right ventricle, but we do not recollect having seen, or read of, an instance where it occurred from the same state of the left ventricle existing alone.

*Case of Paralysis of the Left Arm cured by the application of Leeches to the Anus.* By M. Husson.

A woman, fifty-three years of age, a *French-Billingsgate* porter, of a very strong constitution, very liable to inflammatory affections, perceived one morning, on taking up a burthen, that she had no strength in her left arm: soon afterwards it was entirely paralyzed. She entered the Hotel Dieu: she could not move the arm, and it was insensible to the severest pinches. The functions were in other respects well performed, and she could use her other limbs as well as ordinarily. Eight leeches were applied to the anus. After an abundant evacuation of blood, the patient experienced a tremulous sensation in the affected arm, and in the course of the same day the sense and power of it were perfectly restored.

*An Instance of Death caused by violent Grief.* By M. Husson.

The records of medicine abound with similar cases. Dissection of the body, which might have made this particularly interesting, was not permitted.

*Case of Bloody Flux depending on an Affection of the Liver.* By M. Husson.

This case presents nothing remarkable, with respect to novelty or peculiar interest. It came on after a fall on the right hypochondre against a hard body. It terminated favourably.

*Cases of Peripneumony cured by the Application of a Seton.* By M. Husson.

The consequences of acute peripneumony, somewhat alleviated by venesection, leeches to the chest, and *pectoral potions and drinks,*

consisting in pain in the chest, oppression of breathing, cough with expectoration of mucous matter, in one instance tinged with blood, and hard and frequent pulse, disappeared soon after the passing of a seton into the side corresponding to the seat of pain, in two cases. We every day see similar results from blisters.

*Case of Dropsy cured by Compression joined to the ordinary Means.*
By M. Husson.

This case was apparently similar in respect to its nature and results to two related by Dr. Stoker, and published in the first volume of a production that may be opposed with pride to the *Annuaire Medico-Chirurgicale*, the "Transactions of the Association of the Fellows and Licentiates of the Dublin College of Physicians."

We now arrive at some observations by M. Boyer, first surgeon to La Charité; the first of which is,

*A Case of Wound of the Popliteal Artery cured by Ligature of the Crural Artery.*

The most remarkable thing in this case, to English surgeons at least, is the mode in which M. Boyer tied the crural artery. He says, "I laid bare that artery about the middle of the thigh, and, after having entirely isolated it from the surrounding parts, I passed *four ligatures* under it, by means of a large curved needle. I tied *two* of these ligatures on a piece of sparadrap made of diachylon plaster mixed with gum, rolled up into a cylinder A LITTLE LESS IN SIZE THAN THE LITTLE FINGER; the two other ligatures were left free."

We stop to strike out another *axiom* from the *De la Literature*: "*Les vérités importantes une fois découvertes, frappent tout le monde presque également.*" Madame de Stael herself would acknowledge the validity of the above grounds for it; and we must then notice the other remarkable circumstances in this case. The whole of the four ligatures and the prodigious piece of sparadrap came away on the twelfth day after the operation, and at the end of a *month* there remained only a small part of the wound uncicatrized; in consequence of which the patient was not permitted to leave his bed. Here the history of the case terminates.

The other observations by M. Boyer relate to

*A Case of inconvenient Deformity of the Mouth and Neck, produced by ill-formed Cicatrices, arising from a severe Burning of those Parts.*

This closely resembles one that occurred several years since to the observation of Mr. Nesse Hill, of Chester; and the operation which M. Boyer conceived to be capable of remedying the inconvenience of it, was similar to that performed by Mr. Hill on his patient.

A woman, fifty-six years of age, had her face and the anterior part of her neck severely burned: a foolish practitioner of surgery had suffered the lips to adhere together, so as nearly to close the orifice of the mouth; and a firm cicatrix extended from the chin to the sternum, that retained the head inclined forwards in a very uncomfortable position. The lips were separated by an incision, and the upper one kept removed from the lower by means of silver hooks fixed behind the head. Four transversal incisions, penetrating the whole thickness of the cicatrix, at the distance of half-an-inch from each other, enabled the head to be freely raised up; and it was kept in a proper position by a steel rod, having a flat cross-bar at its upper extremity for the chin to rest on, which was fixed at the front of the body to a sort of corset, and which could be raised or lowered by means of a screw attaching it to the corset. The operation succeeded in a very satisfactory manner.

The above cases are all that M. Boyer has contributed to the *Annuaire*. The rest of its contents will engage our attention at a future time.

---

*On Mediate Auscultation; or a Treatise on the Diagnosis of the Diseases of the Heart and Lungs, principally founded on this new Mean for Exploration.* By R. T. H. Laennec, M.D. &c. &c. Vol. II. Paris, 1819.

[In continuation from page 342.]

*Œdema of the lungs*, is an infiltration of serous fluid in the pulmonary tissue, to such an extent as to diminish in a remarkable degree its perviability to the air. It is but rarely a primitive and idiopathic affection. It occurs most frequently with dropsy of other parts in cachectic subjects, or towards the fatal termination of fevers of long duration; or from some organic affections, and especially those of the heart. Peripneumony, when it terminates by resolution, appears also to leave a great disposition to this malady. Chronic catarrh is equally likely to induce it, and many patients of the disorder just designated die from suffocation produced by the development of œdema of the lungs. It ordinarily takes place within a few hours before death, but it has in many cases continued several weeks, or even months; and in some of these instances it appears to have been an idiopathic affection. It presents the following appearances on dissection: When it occupies the whole of one lung, it is of recent date, the pulmonary tissue is of a pale-grey colour, and devoid of its natural pale roseate hue: its vessels seem to contain less blood than ordinarily. The lung is more heavy and dense than in the healthy state, and does not shrink on the exposure of the cavity of the chest; but it still crepitates, on pressure, almost as sensibly as in the natural condition, though the impression of the finger remains more strongly marked on it. On being cut into, there flows from it an abundance of pellucid serous fluid, nearly colourless, or of a very-pale dun hue. The texture of the air-cells has suffered no apparent change.

The symptoms of œdema of the lungs are very equivocal. The oppression of breathing, a slight cough, and expectoration of a watery fluid, are the only signs which may cause a suspicion of its existence. Percussion does not indicate any thing, both sides being ordinarily affected at the same time; and, when only one lung is œdematized, or is so much more than the other, this mode of exploration does not furnish any evident results.

The stethoscope offers two means of recognizing this state of the lungs. Respiration is heard much less clearly than might be expected, from the efforts by which it is effected, and from the great dilatation of the thorax with which it is accompanied. There is heard at the same time, as in the first degree of peripneumony, a slight crepitation, more like the *rattles* than the sound of healthy respiration. These *crepitant rattles* are less forcible than those perceived in the first degree of peripneumony; but it is hardly possible to distinguish them without the aid of the general signs of the two affections.

We turn from this obscure and comparatively unimportant subject, practically regarded, to one possessing the highest interest; the malady now becoming generally known on the continent by the appellation of *pulmonary apoplexy.* Although of very frequent occurrence, the anatomical characters of this affection have not been at all correctly understood: it has been chiefly regarded in its principal symptom, a copious and abundant *hemoptysis.*

The ancients attributed hemoptysis to hemorrhage immediately coming from a ruptured blood-vessel in the lungs; and this notion, become popular, is still that of those physicians who think it prudent not to admit any new doctrine until it has become so general, that they are at last obliged to admit it, even without examination. Modern anatomists have for several years past proved that the above notion respecting hemoptysis is incorrect. There are, however, two species of hemorrhage from the lungs, which may arise in the way above designated: that which depends on the opening of an aneurism into the trachea, the bronchiæ, or the pulmonary tissue; and that which may be produced by the rupture of a blood-vessel traversing a tuberculous excavation, a case of the utmost rarity, and of which there appears to be no other instance than that related by Bayle. These two species are followed by sudden death, and cannot account for the common form of hemoptysis. Hemoptysis is more commonly attributed to a morbid state of the mucous membrane of the lungs, by which it exhales blood, instead of secreting mucus. This origin of the disease is incontestibly true in some slight cases; such as those which accompany pulmonary catarrh and peripneumony, and that which is owing to the irritation produced by the development of tubercles in the lungs. The last species may, in some instances, be the effect of a real laceration of the engorged pulmonary tissue; especially when it happens at the time when the softened tuberculous matter makes its way into the bronchiæ.

But, the cases of violent and abundant hemoptysis, those which are hardly, or not at all, repressed by venesection and revulsives, depend on a more serious cause; the first effect of which is a considerable alteration of the state of the pulmonary tissue itself. This alteration consists in an induration equal to that of a lung most completely hepatized, but it is in other respects very different from it. The induration is always partial, and does not even occupy a considerable portion of the lungs: its most ordinary extent is from one to four cubic inches. It is always very exactly circumscribed, and, at the point where the induration ceases, the engorgement is as great as in its centre. The surrounding portion of the lungs is quite healthy, and crepitates on pressure, presenting nothing like that state of density gradually becoming less, and disappearing in an indistinct manner, which is found in peripneumony. The lung is, indeed, often very pale round the hemoptysic engorgement; but it is sometimes of a deep-rose or even red colour, and infiltrated, or rather stained, with florid blood: yet, in this case even, the line of demarcation between the dense engorgement and the slight infiltration of blood, is always very distinct. The engorged part is of a deep or blackish red colour, exactly similar to that of a clot of venous blood. The surface of an incision made in it is granulated, as it is in the state of hepatization; but, in other respects, the appearance of those two alterations of texture is quite different. In the hemoptysic engorgement the indurated part presents an homogeneous aspect, and the dark colour of the part permits us to discern no more of the natural texture of the lung than the bronchiæ and the largest order of blood-vessels, the tunics of which have even lost their white colour, and seem to be dyed with blood. If the incised surface be scraped with a scalpel, a little black and half-coagulated blood may be removed from it, but always in less quantity than the bloody serum which oozes from the lung when hepatized in the second degree. The granulations, when held up to the light, appear larger than those found in the state of hepatization. Sometimes the centre of the induration is softened, and occupied by a coagulum of pure blood.

This alteration of the state of the lungs is evidently the result of a sanguineous exhalation into the air-cells of the lungs, and hence it has been considered right to term it *pulmonary apoplexy;* as it resembles in its origin the exhalation of blood in the brain, which produces apoplexy, properly speaking. It is somewhat to be regretted, that the appellation has been here so improperly applied. The term apoplexy designates some of the remote effects of the effusion of blood in the brain, not the state of that organ itself: it is therefore incongruous to apply it to the sanguineous exhalation in the pulmonary tissue.

We sometimes find two or three distinct engorgements in the same lung, and very often both lungs are affected in the same manner. These engorgements are ordinarily situate about the centre of the inferior lobule, or towards the middle of the posterior part of the lung: it is, consequently, in the back, and the lower parts of the chest, that they must be sought for with the stethoscope. It is very often accompanied with hemorrhage really occurring from the mucous membrane of the bronchiæ, and this membrane is, in this case, found to be very red and turgid.

Whatever may be the severity of this affection, the resolution of the engorgement appears to take place with great facility; because a great many persons have perfectly recovered from the most violent hemoptysis, and no trace of the engorgement has been found in their lungs, when they have died a few years afterwards, from some other disease.

The principal symptoms of this malady are, great oppression of breathing; cough, accompanied with considerable irritation of the larynx, and sometimes with severe, and even acute, pains in the chest; expectoration of pure frothy or spumous blood; sometimes, however, this fluid is mingled with saliva and a little mucous from the bronchiæ and fauces. The pulse is frequent, very large, and presents a peculiar sort of vibration, even when it is soft and weak, which it is, ordinarily, after the lapse of a few days.

Of all the symptoms, the spitting of blood is the most constant and severe. It is ordinarily very abundant, and returns, after intervals of cessation, with a sudden cough, oppression, anxiety, either intense redness or extreme paleness of the face, and coldness of the extremities. When the quantity of blood spitten is very abundant, it comes up sometimes with only a slight cough, and is accompanied with a contraction of the diaphragm like that which takes place in the act of vomiting: hence, many patients who have had an abundant hemoptysis say *they have vomited blood.*

Exploration with the cylinder discovers two signs by which this engorgement of the lungs may be known to exist: the first is, absence of the sound of respiration in a very small portion of the lungs; the second, is that of mucous rattles, the bubbles of which appear extremely large, and seem to become dilated as they pass through the bronchiæ, and to burst from excessive distension. The sound from this rupture may be heard in a very unequivocal manner.

The extreme danger attendant on this engorgement, and the possibility of its resolution, should lead to a vigorous treatment of it, by copious blood-letting, as long as it exists, by vesicatories and revulsives. The fear of weakening the patient would be ill-founded in this case, for the most copious bleeding, commonly effected, does not equal the quantity of blood which a patient having this disease often spits in a few minutes.

It is necessary to add, that the hemoptysic engorgement may be suddenly formed, and immediately suffocate the patient, when, as in a case related by CORVISART, death may happen before expectoration of blood could take place.

We pass over the dissertation on *pulmonary catarrh*, from its containing but few observations of striking novelty, individually considered; but we must remark, it furnishes a very excellent history of that disease, when viewed in detail. The mucous rattles constitute the most strongly-marked sign of this affection which is developed by the stethoscope.

We have already given an account of the phenomena perceived by means of the cylinder respecting the action of the heart in the healthy state of that organ: we have now to notice some of its most important diseases, and the means for their diagnosis.

Dilatation and thickening of the ventricles of the heart, are the most common morbid affections of that organ. One or other, or a combination of both of these states, may be produced by great difficulty of breathing, of long continuance, in consequence of the habitual efforts this organ is obliged to make to force the blood through the lungs, in spite of the resistance opposed to its passage by the cause of the dyspnœa. Hence phthisis, empyema, chronic peripneumony, and emphysema of the lungs, produce diseases of the heart; and, from the same reason, those bodily exertions which require laborious efforts, and such as are calculated to hinder free respiration, constitute one of the most common of the remote causes of those diseases.

On the other hand, the diseases of the heart may, from the intimate relation existing between this organ and those of respiration, give rise to various species of diseases of the lungs. They constitute one of the most frequent causes of œdema of the lungs, and hemoptysis; but, when they coincide with chronic pleurisy, phthisis, emphysema, and in general with any chronic disease of the lungs, they will be found, on tracing correctly their history, to have been the consecutive affections. Another cause of dilatation of the heart, with or without hypertrophy or thickening of the parietes of its ventricles, is a disproportion between the volume of the heart and the diameter of the aorta. Any cause obstructing the flow of blood from the heart, will excite this organ to more vigorous contraction; and hence, as is the case with muscles in general, an increase of its bulk will ensue. M. Laennec thinks that a natural want of due thickness of the parietes of the ventricles of this organ, will give rise to a morbid dilatation of them.

Hypertrophy of the heart is not, as some pathologists have supposed, always accompanied with a proportionate augmentation of the volume of the cavities of its ventricles; most frequently, indeed, there is a considerable diminution of their volume, and this portion of the heart presents almost a solid muscular mass: but we need not dwell on these points on the present occasion, as they were considered in the Proëmium to the present volume of this Journal. The columnæ carneæ are often much enlarged, as well as the parieties of the ventricles.

Exploration with the cylinder furnishes very positive and constant results in hypertrophy of the left ventricle of the heart. The contraction of this, when explored between the cartilages at the sternal extremities of the fifth and sixth ribs of the left side, gives a very strong impulse, and a more dull sound than when it is in the natural state: it is more prolonged in proportion to the greater degree of the hypertrophy. The contraction of the corresponding auricle is very short, but little sonorous, and thence hardly sensible in the more severe cases. The pulsations of the heart are heard only in a small extent of space: they are most frequently hardly discernible under the clavicle and the upper end of the sternum. Sometimes they can be heard only where they can be felt, that is to say, between the cartilages of the fifth and seventh ribs. The general signs of this affection are very uncertain and inconstant: this is the case, too, with those of the same affection of the right ventricle; only disordered respiration is here more commonly present. There is however one sign, which had been designated by Lancisi, but thought to be inconstant by Corvisart, that M. Laennec says he has invariably witnessed in cases of hypertrophy of the right ventricle to any considerable extent: this is, swelling of the external jugular veins, accompanied with pulsations analogous to, and isochronous with, those of an artery. This M. Laennec has never seen in hypertrophy of the left ventricle existing alone. The pulsations are ordinarily confined to the lower part of the neck. The signs developed by the stethoscope in hypertrophy of the right ventricle are similar in themselves to those of the same affection of the left ventricles; but here the pulsation is discerned particularly under the inferior part of the sternum.

In a small proportion of cases, both ventricles are simultaneously affected with hypertrophy.

Dilatation of the ventricles of the heart, the *passive aneurism* of Corvisart, presents an enlargement of the cavities with thinning of the parietes. To these characters are ordinarily joined a remarkable softening of their muscular substance, which is also much paler than in the natural state: the colour of it is sometimes even yellowish. Both ventricles are most frequently affected at the same time. Corvisart states the signs of this affection to be, "a soft and weak pulse; feeble, dull, and sinking, palpitations; the hand feels a soft body raise up the ribs, but which does not strike them with a sharp, clear, blow; it seems as if the pulsations were weakened by forcible pressure." These signs are, however, both uncertain and inconstant. Portal relates a case in which the pulse continued hard and strong until just before death. The action of the heart cannot in many instances be at all perceived by the hand when applied to the chest, and the perception of the rising of the soft body we believe to be a mere thing of the imagination.

M. Laennec says, the only certain sign of dilatation of the left ventricle, is that developed by the stethoscope, which is the clear and rattling sound of the contraction of the heart heard between the cartilages at the sternal ends of the fifth and seventh ribs of the left side. The degree of clearness of this sound, and its extent, form the measure of the dilatation: thus, when the sound of the contraction of the ventricle is as clear or acute as that of the contraction of the auricle, if the action of the heart be at the same time heard in the back towards the right side, the dilatation is extreme. The sign of the same affection of the right ventricle, developed by the cylinder, is a sharp or rattling sound from the contraction of the heart, heard under the lower part of the sternum, or in the space comprised between the cartilages of the fifth and seventh right ribs.

When dilatation of the ventricles of the heart accompanies hypertrophy, the signs developed by the stethoscope will present a combination of those just designated.

*Dilatation of the auricles of the heart*, is a case of very rare occurrence. Sometimes, however, it exists with hypertrophy or dilatation of the ventricles; and, still more rarely, the auricles are dilated when the ventricles are in the natural state.

Diversities in the volume of the four cavities of the heart often exist after death, solely as a consequence of the manner in which life has ceased; but, in the natural state, they are very nearly equal in the living body.

M. Laennec has never found evident dilatation of the auricles without some degree of thickening of them; and, on the other hand, he has never seen hypertrophy of them without augmentation of the capacity of their cavities.

The most common apparent cause of dilatation of the left auricle, is contraction of the orifice by which it communicates with the cavity of the corresponding ventricle, from cartilaginous or osseous induration of the mitral valve, or excrescences formed on its surface. This state of the valve is sometimes attended with permanent retraction of it, and consequently a permanence of the aperture between the auricle and ventricle; and dilatation of the auricle may happen in this case from the distensive impulse of the ventricle during its contraction. He has never observed dilatation of the auricles without alteration of the structure of their valves. Dilatation of the right auricle takes place most frequently after hypertrophy of the right ventricle. Diseases of the lungs, which M. Corvisart places amongst the ordinary causes of this dilatation, appears to M. Laennec to give rise most frequently merely to a distension of it in the dead body. He has not yet been able to examine these affections sufficiently to determine any thing with precision respecting their signs: but it appears that they are liable to be confounded with those of dilatation of the ventricles.

M. Laennec notices *induration*, *softening*, and *atrophy*, of the muscular structure; but he does not adduce any original and novel observations of much importance respecting those affections. The *fatty degeneration of the heart*, is so interesting a malady, that we must adduce his principal remarks on this subject; for, though they may supply us with no addition to our knowledge of this subject, it is interesting to know what a pathologist of M. Laennec's talents and research has observed of the nature and origin of the affection above designated.

The most remarkable instance of this species of morbid alteration of the structure of the heart that medical records present, is that observed by Dr. Cheyne; an account of which was inserted in a late Number of this Journal, from the Dublin Hospital Reports.

M. Laennec says, he has many times found, in subjects who had died of different diseases, hearts surcharged with fat, which, deposited between the muscular substance of the heart and its pericardium, which is ordinarily intimately united with it, was principally accumulated about the point of union of the auricles and ventricles, along the trunks of the coronary vessels and the two margins of the heart, at its point, and at the origin of the aorta and pulmonary artery. Sometimes the posterior surface, or that corresponding to the right ventricle, is equally affected by it throughout almost the whole of its extent: on the contrary, the surface of the left ventricle but rarely presents any considerable quantity.

The more a heart is surcharged with fat, the less, in general, is the thickness of its parietes; sometimes even this thickness is reduced almost to nothing in some points: this is particularly the case at the point of the ventricles and at the posterior part of the right ventricle. If these parts are examined by looking at them from within the ventricles, they present the natural aspect; but, if they are cut into from without inwardly, we arrive at the cavity without having encountered hardly any muscular structure; and the columnæ carneæ of the ventricles, as well as their pillars, appear to be attached together, in those points, only by the internal membrane of the ventricles.

The fat, M. Laennec thinks, does not, in these cases, appear to be the product of a degeneration of the muscular substance of the heart, because it can be separated from it by dissection: sometimes, however, layers of fat are deeply insinuated between the packets of muscular fibres; but, even in this case, the two substances are precisely and remarkably distinct from each other, and not the slightest shade of colour or of structure confounds them. It is, then, very probable, that, by means of pressure, or by some derangement of nutrition, the muscular substance of the heart has been lost in proportion to the formation of the fat which envelops it.

There is very often found, with the above-mentioned appearances, a large quantity of fat accumulated in the lower part of the mediastinum, and especially between the pericardium and the pleura. This fat blocks up, and runs into, a great number of small blood-vessels, which gives it a reddish colour: these portions of fat are afterwards, in some instances, forced out of the vessels, and drive the pleura before them, so that they are enveloped by that membrane, and they project into its cavity under the form of irregular fringes, which have a sort of gross resemblance to the comb of a cock.

M. Laennec has never met with a case of real degeneration of the muscular substance of the heart into fat, excepting in a very small part of it near its apex.

We pass over the author's remarks on some other of the diseases of the heart, from their not relating to original observations of important novelty; the abstract we have given of these alone has already extended beyond what we could have desired. The same remarks will also apply to what relates to aneurism of the aorta. The phenomena developed by the stethoscope are very equivocal and inconstant in the affections just alluded to. Aneurism of the aorta has not yet been sufficiently examined by the aid of the stethoscope, to enable him to determine precisely the utility of it on this occasion; but simple pulsations, (that is to say, without contraction of the muscles,) isochronous with the pulse of the arteries, and a strong impulse, beyond the precordial region, in the course of the aorta, may give rise to a suspicion of the existence of the disease just named.

Here we terminate our analysis of this work; not very well satisfied with our labours on this occasion, when we perceive how much interesting original matter we have left unnoticed in our course through it, notwithstanding the length of our abstract and our attempts at conciseness; but, we think, the greater part of the more studious cultivators of medicine will peruse the original: and we have produced what may give an idea of the merit of the work, and indicate the respect that is due to its author by all those who have a due love for medical science.

### *On the Means for arriving at the Bladder by the Rectum; and on the advantages and inconveniences attached to this Method for extracting Stones from the Bladder: with some Cases in support of this Operation.* By L. J. SANSON, Doctor in Medicine of the Faculty of Paris, House-Surgeon at the Hotel Dieu, &c. &c. 4to. pp. 50. Paris, 1817.*

Etiam et tentasse decorum.

THE history of the case inserted in the present number of this Journal, in which Dr. Barbentini put in practice the method of cutting for the stone proposed by M. Sanson, has again directed our attention to the dissertation before us; and we have considered that an abstract of the most useful part of its contents will prove interesting to the generality of our readers, to whom the proposition of M. Sanson may not be sufficiently well known.

On examining attentively the several methods of cutting for the stone that have been in use, and on reflecting on the importance of the parts which may be interested by each of them in particular, we may be easily convinced, says M. Sanson, that the most secure way of arriving at the bladder amongst those which have been resorted to, is that of JOHANNES DE ROMANIS; and it seems to be only necessary to make a larger wound than is directed by Marianus Sanctus, and to divide by *incision* the parts which he tore, to avoid all the inconveniences attached to his operation, and to render it preferable to any of those in use. A demonstration of the anatomical relations of the parts concerned in it, will, apparently, render evident the propriety of the above remark.

The rectum, regarded in its totality, is extended from the superior aperture of the pelvis to the anus: it is at first directed a little obliquely from left to right, and from above downwards; it is curved towards the lower part of the cavity of the pelvis, so as to be directed from behind forwards under the bladder, as far as a level with the prostate gland, below which it forms a new curve, by which it is directed a little backwards as it continues its course in a downward direction.

Considered under the point of view which now occupies our attention, it may be considered to be formed of three parts distinct from each other by their situation, their structure, and by the nature and importance of their connexions: those parts are marked out by the several curves which have been described.

The first, or superior portion of the rectum, comprising more than half its length, is free, flexible, covered by the peritoneum, and attached in a loose manner to the posterior paries of the pelvis by a fold of that membrane, and has only general and variable relations with the sacrum and bladder, and such as are not of importance in our present view.

---

* *Des Moyens de parvenir a la Vessie par le Rectum, avantages et inconveniences attachés à cette Méthode pour tirer les Pierres de la Vessie; avec des observations a l'appui.* Chez Mequignon-Marvis, Paris.

The second or middle portion, comprised between the two curves of the intestine, is about three inches in length; its direction is obliquely downwards and forwards in a slight curve; it is fixed in its relative position, and corresponds, backwardly, to the lower part of the sacrum, to the coccyx, and to the floor formed by the ischio-coccygean muscles; in front, with the *bas-fond* of the bladder, from which it is separated outwardly and below, by the vesiculæ seminales and the vasa deferentia, and yet more inferiorly, by the prostate; its sides correspond to an abundant mass of cellular texture. The structure and organization of this, differ considerably from that of the first portion: 1°, in its being entirely devoid of peritoneum, excepting the anterior surface of its most superior part in the case of considerable retraction of the bladder; 2°, in its muscular coat being much thicker, and formed of longitudinal fibres, much stronger, and more numerous; 3°, in its being completely environed by a loose and very abundant cellular tissue, excepting below the prostate, where this tissue is dense: this permits of the great variation of volume which it occasionally assumes.

The third and last portion of the rectum commences just on a level with the prostate, and ends at the anus; its length commonly varies from an inch to an inch and a half; it is larger in diameter at its superior than at its inferior part. Near its origin it is surrounded by an abundant cellular tissue, except in front, where it corresponds to the prostate. In all the rest of its extent, it is enveloped by the sphincters. Its structure is, then, very different from that of the other portions; the muscular coat of the former portion terminates suddenly at the commencement of this; the mucous membrane is alone continued on to the skin, surrounded by the circular muscular fibres belonging to the sphincters, which form, by their assemblage, a sort of ring, much thinner at its origin than near the skin, where it becomes very thick, and gives origin to two prolongations in form of tails, the anterior of which is directed towards the bulb of the urethra, whilst the posterior one runs to the coccyx.

The two latter portions, then, form, together, a length of intestine of at least four inches, wholly surrounded by an abundance of cellular texture, subject to be irritated by the varieties of fecal matter and the foreign bodies they often contain, and which is every day operated on by surgical instruments without much danger, sometimes throughout a considerable portion of its extent, and indifferently in any point of its circumference, not excepting its anterior and middle portion, which, from its intimate relation with the *bas-fond* of the bladder, has been chosen as the medium for tapping the bladder in cases of retention of urine, where the catheter cannot be introduced.

The posterior and inferior portion of the bladder, termed its *bas-fond*, extended forwards from the recto-vesical layer of the peritoneum to the origin of the urethra, continued into the sides of the organ without any line of demarcation, of nearly equal dimensions in every direction, is united by firm adhesions to the uterus, the vasa deferentia, and to the vesiculæ seminales, which, traversing it obliquely from behind forwards, and from without, inwardly, divide it thus into three surfaces, two of which are lateral, convex, larger forwards than backwards, placed without the vesiculæ seminales, and correspond to an abundant fatty cellular texture, which separates them from the levator ani; whilst the third and middle surface, placed between the two vesiculæ seminales, of a triangular figure, having a base turned backwards which corresponds to the peritoneum, and a summit directed forwards which corresponds to the prostate, is immediately opposite to, and in contact with, the middle portion of the rectum, the curve of which it exactly follows as far as the prostate gland; then it separates from it, to take an oblique direction from behind forwards and a little upwards, as far as the origin of the urethra. This, embraced by the prostate and confounded with the neck of the bladder, is not near so close to the symphisis pubis as it has been believed, since, placed on a line extending from the lower part of this symphisis to the point of the coccyx, it is about two inches distant from it. The skin of this region and the anterior prolongation of the sphincter of the anus, the canal of the urethra in front, and the last portion of the rectum, having the same sphincter extended behind it, form the three sides of a triangular space filled with fatty cellular texture.

If, taking the rectum as the point of departure, we examine the parts situate in front of the intestine in the order in which they present themselves, we find, 1°, proceeding from the middle portion of the rectum a little forwards and downwards, a layer;—more or less thick, of loose cellular texture, containing a net-work of small veins, the bas-fond of the bladder, and the cavity of this organ: 2°, proceeding from the curve formed by the intestine below this region in a direction towards the anus, and taking a more nearly horizontal direction;—a layer of thin and close-set cellular texture, and the prostate traversed by the urethra: 3°, proceeding from the most inferior portion of the intestine, and following a horizontal line running forwards;—the sphincter of the anus, the triangular space above described, and the bulb of the urethra.

From whatever point we proceed, following this direction, we find no vessel, excepting the capillaries serving as anastomoses between those of one side to the other.

On considering these relations, M. Sanson perceived that, on making an incision in the sphincter ani from the rectum towards the root of the penis, he should lay bare not only the point of the prostate, but also a more or less considerable portion of its inferior surface, and that he should then be able to penetrate the cavity of the bladder, either by its neck on traversing the prostate, or by its bas-fond on running along its posterior part. It was the second mode he first determined to try on the dead body.

He placed the body in the ordinary position for the common operation; a grooved sound was introduced, and confided to an assistant, who was desired to hold it in a perfectly-vertical direction; the fore-finger of the left hand was passed into the rectum, with its palmar surface upwards; a common bistoury, lying flat, was glided along this surface, and, after having turned its cutting margin upwards, it was made to divide, at one stroke, in the direction of the raphé, the external sphincter of the anus and the lower part of the rectum surrounded by it. The inferior surface of the prostate was laid bare; the finger was then passed along this surface until it arrived behind the gland; he then recognized, through the rectum and the bas-fond of the bladder lying close together, the sound, which the assistant had all along maintained in the same position: he plunged the point of the bistoury into this part, and, guided by the groove of the sound, he made an incision in it of about an inch in length: the urine passed by the wound.

The operation being thus completed, and the body maintained in the same position, an examination was made of the state of the parts engaged in it. This displayed: At the superior part of the anus, a wound dividing the external sphincter throughout almost the whole of its thickness, and at the bottom of this wound a nearly-vertical incision, through which the interior of the bladder could be easily and distinctly seen; and, below this wound, the anus opened to a considerable extent, in consequence of the division of its sphincter.

The bladder, seen from its interior, presented an incision commencing immediately behind its neck, and which extended, following exactly the median line, as far as the middle of the space which separates the orifices of the ureters.

The fibres of the spincter, the lowest portion of the rectum, the most posterior part of the prostate, and the bas-fond of the bladder, had been alone interested.

The objections which immediately present themselves to this operation, is the danger of a fistulous communication between the cavities of the bladder and rectum, and the ill consequences from the passing of the feces into the former, before the healing of the wound. These M. Sanson endeavours to obviate, by cases in which those cavities had, from several accidents, been interested in the same way as they are in consequence of this operation, and in which no such fistulæ were formed, nor did any serious inconvenience result from the passing of feces into the bladder. The case of Dr. Barbentini is a still better argument.

The peculiar advantages of this operation are:

1. The facility and promptitude with which it may be performed; two strokes of a bistoury being sufficient to complete it.

2. The little danger attendant on the lesion of the parts interested.

3. The little depth of the wound, which permits us to see into the cavity of the bladder, and hence the comparative facility with which stones may be extracted.

4. The certainty of avoiding hemorrhage, no considerable essel existing in the median line, and, in the case of the presence of such a vessel in deviations from the ordinary structure, a ligature might be easily applied to it.

5. The certainty of avoiding incontinence of urine and inflammation of the prostate, this gland and the neck of the bladder being avoided.

6. The possibility of extracting stones of very great size through the largest part of the inferior aperture of the pelvis.

These advantages are positive, and several of them of considerable importance; and, unless practice shall prove, what theory cannot anticipate, that they are counterbalanced by disadvatages, the operation in question must be regarded as preferable to that of Cheselden. Whether or not it will be fairly submitted to the test of modern practice, is another point; experience seems to have proved the superiority of the high operation to the lateral one, but it appears a doubtful matter whether or not it will again come into general use.

Another way of penetrating the cavity of the bladder is, by proceeding as in the operation above described, as far as the completion of the *first incision;* and then, instead of commencing the second incision behind the prostate, effecting it below and anteriorly to this gland, the bistoury being directed towards the groove of the staff, we divide, along the median line, the prostate, the prostatic portion of the urethra, and the neck of the bladder, in which we may make an incision from twelve to fifteen lines in length without again touching the rectum. We then cut all the parts which were torn by the method of Marianus, without touching those which he cut. The most serious injuries effected in his operation are thus avoided; but there is the danger of those which result from the division of the neck of the bladder.

This operation wants, besides, several advantages of the former operation of Sanson; as, the possibility of seeing clearly into the cavity of the bladder, in lean subjects, and the great and free extent of space that it affords for the extraction of the stone.

